# Supplementary material for: EEG Dynamics of Error Processing and Associated Behavioral Adjustments in Preschool Children
Source: Brain Sci. 2023 Mar 29;13(4):575. doi: 10.3390/brainsci13040575 (PMC10136969; doi:10.3390/brainsci13040575)
Supplement: Supplementary file 1 [file brainsci-13-00575-s001.zip › Piettoetal_supplemental_material_S1.pdf]

Supplemental Material S1

Sociodemographic Scale

The scale included variables related to Unsatisfied Basic Needs (UBN) indicators. A home was considered to have UBN if at least one of the following indicators was identified: (1) inappropriate dwelling (at least one of the following characteristics: type of dwelling - Room tenancy / Hotel / Street; Type of floor - Earth; Water access - Well water / River / Canal; Type of bathroom - Own bathroom without toilet / Shared bathroom/ No Bathroom; Type of roof - Cane / Wood; External walls - Cardboard; Dwelling property - Occupied); (2) overcrowding (more than three people per room); (3) head of household with incomplete secondary schooling with more than four dependents; (4) presence of school-aged children not attending any educational system; (5) absence of waste discharge system in the household; (6) absence of water supply pipes inside the house or land, and (7) living unfavored neighborhoods (e.g., slums). This criterion was met whether the name of the neighborhood was included in the city's registry of vulnerable neighborhoods, if it was required to pay property or council taxes, or if there were social service organizations working in it. These criteria were chosen following methodologies implemented by the Argentine National Institute of Statistics and Census (INDEC, 2004).
