# Supplementary material for: EEG Dynamics of Error Processing and Associated Behavioral Adjustments in Preschool Children
Source: Brain Sci. 2023 Mar 29;13(4):575. doi: 10.3390/brainsci13040575 (PMC10136969; doi:10.3390/brainsci13040575)
Supplement: Supplementary file 1 [file brainsci-13-00575-s001.zip › Piettoetal_supplemental_material_S2.pdf]

Supplemental Material S2

Normality and Homogeneity analyses of behavioral and neural variables

Table S2 1.

Normality and Homogeneity analyses for behavioral measures

	Normality			Homoscedasticity			
	SW	***df***	***p***	***L***	***df1***	***df2***	***p***
Correct-trial RT	.992	91	.876	6.69	1	180	.010
Error-trial RT	.977	91	.110				
Post-correct slowing	.990	91	.731	42.51	1	180	<.001
Post-error slowing	.992	91	.874				
Post-correct accuracy	.969	91	.030	3.21	1	180	.075
Post-error accuracy	.978	91	.133				
*Correct-trial RT**	.980	91	.178	15.57	1	179	<.001
*Error-trial RT**	.991	90	.816				
*Post-correct slowing**	.800	91	<.001	.04	1	179	.838
Post-error slowingT	.970	90	.038				
*Post-correct accuracy**	.964	91	.012	2.41	1	179	.122
*Post-error accuracy**	.971	90	.045				
RSI - 1st tertile	.980	66	.357				
RSI – 2nd tertile	.953	66	.013	9.54	2	195	<.001
RSI – 3rd tertile	.939	66	.003				
PEA – 1st tertile	.980	66	.370				
PEA – 2nd tertile	.969	66	.099	.37	2	195	.691
PEA – 3rd tertile	.928	66	.001				
RSI_T - 1st tertile	.974	66	.179				
RSI_T – 2nd tertile	.923	66	.001	25.71	2	195	<.001
RSI_T – 3rd tertile	.911	66	<.001				
PEA_T – 1st tertile	.972	66	.149				
PEA_T – 2nd tertile	.964	66	.054	.80	2	195	.449
PEA_T - 3rd tertile	.920	66	<.001				

Note.

SW = Shapiro-Wilk

L = Levene

* = denotes transformed variables. RT and accuracy variables were transformed using natural log and square root, respectively

Table S1 2.

Normality and Homogeneity analyses for neural measures

	Normality			Homoscedasticity			
	SW	***df***	***p***	***L***	***df1***	***df2***	***p***
ERN – 1st tertile	.982	58	.563				
ERN – 2nd tertile	.970	58	.157	2.09	2	171	.127
ERN – 3rd tertile	.992	58	.961				
THETA – 1st tertile	.983	58	.601				
THETA – 2nd tertile	.982	58	.529	.16	2	171	.774
THETA – 3rd tertile	.982	58	.530				
RSI - 1st tertile	.978	58	.378				
RSI – 2nd tertile	.951	58	.021	8.63	2	171	<.001
RSI – 3rd tertile	.951	58	.021				
*RSI – 1st tertile**	.969	58	.145				
*RSI – 2nd tertile**	.926	58	.002	25.83	2	171	<.001
*RSI – 3rd tertile**	.927	58	.002				

Note.

SW = Shapiro-Wilk

L = Levene

* = denotes transformed variables. The variables were transformed using natural log
