# Supplementary material for: EEG Dynamics of Error Processing and Associated Behavioral Adjustments in Preschool Children
Source: Brain Sci. 2023 Mar 29;13(4):575. doi: 10.3390/brainsci13040575 (PMC10136969; doi:10.3390/brainsci13040575)
Supplement: Supplementary file 1 [file brainsci-13-00575-s001.zip › Piettoetal_supplemental_material_S3.pdf]

Supplemental Material S3

Number of trials included in the study

Table S3 1.

Number of trials for each variable used in the analysis

Behavioral measures	**Median**	**Min**	**Max**
Correct-trial RT (ms)	326	46	503
Error-trial RT (ms)	67	8	177
Post-correct slowing (ms)	39	7	129
Post-error slowing (ms)	39	7	129
Post-correct accuracy (%)	39	7	129
Post-error accuracy (%)	39	7	129
Neural measures	**Median**	**Min**	**Max**
Correct-related activity	311	45	497
Error-related activity	60	8	169
Error-related activity before a correct response	45	11	84
Error-related activity before an error response	12	7	39
Error-related activity before a correct response 1st tertile	15	7	29
Error-related activity before a correct response 2nd tertile	14	7	27
Error-related activity before a correct response 3rd tertile	14	8	34
