# Supplementary material for: EEG Dynamics of Error Processing and Associated Behavioral Adjustments in Preschool Children
Source: Brain Sci. 2023 Mar 29;13(4):575. doi: 10.3390/brainsci13040575 (PMC10136969; doi:10.3390/brainsci13040575)
Supplement: Supplementary file 1 [file brainsci-13-00575-s001.zip › Piettoetal_supplemental_material_S4.pdf]

Supplemental Material S4

Comparisons between error-related theta activity before an error recovery with different post-error slowing and before a failed error recovery

We examined whether the error-related theta activity before an error recovery in trials with small, middle or large post-error slowing was different from the error-related theta activity before a failed error recovery. For this purpose, we used the theta powers related to each subject's error that were followed by hits and false alarms. The theta powers that were followed by a hit were grouped into tertiles based on their PES, whereas the theta powers that were followed by a false alarm were grouped into a single group. Then, we computed the area of response-locked waveforms within the time-window: -350 ms - 450 ms. Finally, we used the Wilcoxon signed-rank test to perform pairwise comparisons to evaluate differences in power between error-trials that were followed by correct responses at multiple slow down levels (hits at different PES tertiles) and errors-trials that were followed by another error response (i.e., false alarm).

Thirty-nine subjects who had at least seven error trials in each of the four conditions were included in these analyses. Results yielded that the error-related theta power before another error response was significantly different (significance level after Bonferroni correction $p = .0167$) than error-related theta power of trials with low ($Z = 2.47$, $p = .014$, $r = .40$) and high ($Z = -3.14$, $p = .002$, $r = .50$) levels of PES preceding a correct response. Whereas theta power before a failed error recovery was not different from theta power of trials with middle levels of PES ($Z = 1.07$, $p = .283$, $r = .17$) before recovery from an error.
